# Oncologic Safety of Preoperative Controlled Ovarian Stimulation for Fertility Preservation Among Women with Estrogen Receptor Positive Breast Cancer

**DOI:** 10.1245/s10434-026-20156-8

**Published:** 2026-07-05

**Authors:** Jennifer H. Chen, Irene Peregrin-Alvarez, Carla L. Warneke, Laurie J. McKenzie, Terri L. Woodard, Helen M. Johnson

**Affiliations:** 1 Department of Breast Surgical Oncology, The University of Texas MD Anderson Cancer Center, Houston, TX USA; 2 Department of Gynecologic Oncology and Reproductive Medicine, The University of Texas MD Anderson Cancer Center, Houston, TX USA; 3 Department of Obstetrics and Gynecology, Baylor College of Medicine, Houston, TX USA; 4 Department of Biostatistics, The University of Texas MD Anderson Cancer Center, Houston, TX USA

**Keywords:** Ovarian stimulation, Fertility preservation, Oncofertility, Breast cancer, Estrogen receptor positive, Neoadjuvant, Survivorship

## Abstract

**Background:**

The oncologic safety of preoperative controlled ovarian stimulation (COS) for fertility preservation in women with an in situ estrogen receptor positive (ER+) breast cancer is unclear. The purpose of this study was to compare oncologic outcomes of women with ER+ breast cancer undergoing preoperative COS versus (1) postoperative COS or (2) matched controls not undergoing COS.

**Patients and Methods:**

This was a single-institution retrospective cohort study of women with ER+ breast cancer receiving oncofertility counseling who pursued COS from 2014–2024. The primary outcome was progression-free survival (PFS). A subcohort of patients who received neoadjuvant systemic therapy was compared with a matched cohort of similar patients with ER+ breast cancer who did not undergo COS. Propensity score matching criteria were age, HER2 receptor status, and clinical stage.

**Results:**

Among 51 women with ER+ breast cancer undergoing COS, the median follow-up was 5.5 years. 5-year PFS was similar for preoperative COS (*n* = 32) versus postoperative COS (*n* = 19): 94.1% (95% CI 65.0–99.2%) versus 93.3% (95% CI 61.3–99.0%), *p* = 0.73. Patients undergoing neoadjuvant systemic therapy (NST) and preoperative COS (*n* = 30) experienced a significantly longer time from diagnosis to NST initiation than propensity-score matches who did not undergo COS (48 versus 29.5 days, *p* < 0.0001), but disease-free survival was similar: 94.1% (95% CI 65.0–99.2%) versus 93.3% (95% CI 75.9–98.3%), *p* = 0.15.

**Conclusions:**

In this study of women with ER+ breast cancers, preoperative COS was not associated with increased risks of disease progression or death. While COS was associated with a modest delay in NST initiation, PFS was similar to propensity-score matched patients who did not undergo COS. Results support the oncologic safety of COS in the setting of an in situ ER+ tumor and provide much-needed evidence for young women with breast cancer receiving gonadotoxic NST who desire future childbearing during survivorship.

**Supplementary Information:**

The online version contains supplementary material available at https://doi.org/10.1245/s10434-026-20156-8.

Annually, more than 300,000 new cases of invasive breast cancer are diagnosed and up to 5–7% of these are in women of reproductive age.^1^^,^^2^ Current societal guidelines from the American Society of Clinical Oncology and European Society for Medical Oncology recommend all patients of childbearing age receive comprehensive oncofertility counseling prior to initiation of cancer treatment, particularly gonadotoxic systemic therapy.^3,4^ However, despite these recommendations, studies reveal that both physicians and patients are hesitant to offer and accept fertility preservation due to fear of breast cancer progression and potentially increasing the risk of future disease recurrence.^5,6^

Cryopreservation of oocytes and/or embryos with controlled ovarian stimulation (COS) is the first-line fertility preservation (FP) option for premenopausal women.^7^ Ideally, cryopreservation is performed prior to the start of systemic therapy with COS for an approximate 2-week period prior to oocyte retrieval.^7^ The oncologic safety of COS in estrogen receptor positive (ER+) breast cancer after surgical resection is well established.^7–15^ However, data are limited on the safety of COS before surgery in the setting of a hormone-responsive tumor in situ. During COS, estradiol may reach supraphysiologic levels over 2000 pg/mL, far greater than the average physiologic peak of approximately 200–350 pg/mL during normal menstrual cycles.^16^ Intuitively, the mitogenic impact of high estrogen levels in the presence of an estrogen-responsive tumor may theoretically promote disease progression and lead to cancer cell dissemination through seeding of distant organs.^17^

To date, no studies have specifically evaluated the oncologic safety of preoperative COS with an in situ ER+ breast tumor. Prior studies examining the safety of FP in patients with breast cancer utilized heterogeneous cohorts with limited numbers of patients with ER+ disease and/or treated with neoadjuvant systemic therapy (NST).^11,13,18–20^ As such, the definitive risks of supra-physiologic estrogen levels in the setting of an in situ estrogen-responsive breast cancer on tumor progression, treatment response, and long-term outcomes remain underexplored.

This study aims to determine whether fertility preservation with COS in the preoperative setting is associated with increased risk of progression or mortality in women with an in situ ER+ breast cancer. Specifically, we aimed to compare oncologic outcomes of women with ER+ breast cancer undergoing preoperative COS versus (1) women with similar tumors undergoing postoperative COS or (2) matched controls not undergoing COS.

## Patients and Methods

### Study Design and Cohort Selection

This was a retrospective cohort study of patients with ER+ primary breast cancer who opted to pursue COS after receiving oncofertility counseling at a comprehensive cancer center from 2014 to 2024. Patient demographics, clinicopathologic, treatment, and fertility preservation characteristics, along with survival outcomes, were abstracted from the electronic medical record. Exclusion criteria included receipt of FP prior to breast cancer diagnosis, receipt of breast cancer treatment at an outside institution, and loss to follow-up. An additional cohort of patients with ER+ primary breast cancer who received NST but did not undergo COS was obtained from our institutional database to conduct propensity score matching as detailed below. This study was approved by the institutional review board (Protocol 2024-0508).

### Controlled Ovarian Stimulation Protocols

All patients who underwent COS for oocyte and/or embryo cryopreservation were included in the fertility preservation cohort. COS occurring prior to surgical resection in the neoadjuvant setting was defined as preoperative COS while that occurring after surgery in the adjuvant setting was defined as postoperative COS. All patients receiving neoadjuvant systemic therapy underwent COS prior to receipt of gonadotoxic systemic therapies. COS consisted of both traditional early follicular phase stimulation and random start protocols. All patients received recombinant follicle-stimulating hormone at a total dose ranging from 1425 to 7800 IU (typical range ~2000–6000 IU), with or without the addition of human menopausal gonadotropins at individualized doses based on ovarian reserve and clinical characteristics. Human chorionic gonadotropin was used as the standard trigger, with substitution or addition of concomitant leuprolide in patients with increased risk of ovarian hyperstimulation syndrome. Trigger shots were administered once adequate follicular development was achieved to stimulate final maturation of oocytes. Ultrasound-guided transvaginal oocyte retrieval was performed 34–36 h after trigger administration. Outcomes of oocyte retrieval, subsequent in vitro fertilization if applicable, and the number of oocytes and embryos cryopreserved were recorded.

### Outcomes

The primary outcome was progression-free survival (PFS), defined as the time from diagnosis until first documented disease progression or death from any cause, whichever occurred earlier. Patients who were alive and disease-free were censored at the date of their last disease assessment. The secondary outcome was disease-specific survival (DSS), defined as the time from diagnosis until a breast-cancer-related death. Patients alive at the end of follow-up were censored at the date of their last disease assessment. No deaths occurred during follow-up that were not breast cancer related. Treatment response in the neoadjuvant setting was determined by presence or absence of pathologic complete response (pCR) and percent change in tumor volume, which was calculated from tumor dimensions on pretreatment versus posttreatment imaging reports.

### Propensity Score Matching

To further study the subcohort of patients undergoing preoperative COS and NST, propensity score matching was applied in a 1:1 ratio to select a cohort of patients with ER+ breast cancer who received NST but did not undergo COS.^21^ Cohorts were matched on variables selected on the basis of clinical plausibility and included: age at diagnosis (<  34 years versus ≥ 34 years), HER2 (human epidermal growth factor receptor 2) receptor status, and clinical stage (I–II versus III, American Joint Committee on Cancer 8th edition). Greedy nearest neighbor matching without replacement was implemented using SAS PROC PSMATCH. Propensity scores were estimated using logistic regression, assessing similarity with the logit of the propensity score and applying a caliper width that restricted matching to those within 0.2 times the pooled estimate of the standard deviation of the propensity scores.^22^ In the institutional database of patients with ER+ breast cancer who underwent neoadjuvant chemotherapy, 99 fell within the support region. Matching was limited to one control per case. Matching balance was assessed using visual diagnostics (Fig. S1) and metrics, including standardized mean differences and variance ratios. The total absolute difference in the logits of the propensity scores for all matches was 1.46, and the standardized mean difference in the logit of propensity scores was − 0.001. All absolute standardized mean differences for the covariates were less than 0.1, indicating good balance.^23^ The treated-to-control variance ratios were 0.96 for the logit propensity score and 0.84 for age, both close to one as recommended.^23–25^

### Statistical Analysis

Demographic, clinicopathologic, and treatment characteristics were summarized using frequencies and percentages for categorical variables, means and standard deviations for normally distributed continuous variables, and medians, quartiles, and ranges for continuous variables departing from a normal distribution. The analysis included two sets of comparisons: (1) comparison of women who underwent FP preoperatively versus postoperatively and (2) comparison of the subcohort of women who underwent NST and preoperative FP versus propensity score matches who underwent NST but did not undergo FP. Continuous variables were compared using the two-sample* t*-test, Wilcoxon rank-sum, or Mann–Whitney *U* test, and categorical variables were compared using Fisher’s exact test. For any categorical variables indicating statistically significant differences, a post hoc adjusted standardized Pearson residual analysis was conducted to identify specific categories contributing to the overall test results, with standardized residual greater than 1.96 indicating categories that had statistically significant differences. Testing for differences between the preoperative FP cohort versus the matched cohort used complete case analysis (i.e., a matched pair was included only if both members of the pair had a non-missing value).

The Kaplan–Meier method was used to estimate the 5-year PFS rate and 10-year DSS with corresponding 95% confidence intervals and to evaluate the equality of the survival curves. The log-rank test was used to compare survival curves for the preoperative versus postoperative fertility preservation groups, and the log-rank test stratified by matched pair was used to compare survival curves for the preoperative fertility preservation group versus matched controls. Median follow-up time was calculated using the reverse Kaplan–Meier method. All analyses were conducted using SAS for Windows version 9.4 (SAS Institute, Cary, NC, USA), with *p*-values less than 0.05 considered statistically significant.

## Results

### Baseline Patient Characteristics

Of 65 patients with ER+ breast cancer who received oncofertility counseling and COS during the study period, 14 were excluded: 9 received cancer treatment after FP at an outside institution, 2 underwent FP prior to breast cancer diagnosis, 2 were lost to follow-up, and 1 underwent post-NST FP. Of the 51 patients comprising the final cohort, 32 (62.75%) underwent preoperative FP and 19 (38.25%) underwent postoperative FP (Table 1). The mean age was 34.0 years (SD 4.8) and approximately half (29, 56.9%) were non-Hispanic white. The majority of patients had invasive breast cancer while two (3.9%) had ductal carcinoma in situ. Most patients with invasive disease had ductal histology (45, 88.2%), clinical stage I–II (41, 83.7%), and high-grade disease (28, 60.78%). All patients had ER+ disease, with 84.3% (43) progesterone receptor (PR)-positive, and 42.9% (21) HER2-positive. Patients who underwent preoperative versus postoperative FP had higher rates of ductal histology (96.9% versus 73.7%, *p* = 0.03), clinically node-positive disease (50% versus 16%, *p* = 0.02), high-grade disease (75.9% versus 35.3%, *p* = 0.01), and HER2-positive disease (56.3% versus 17.7%, *p* = 0.01) (Table 1).

**Table 1:** Baseline patient and tumor characteristics, by timing of fertility preservation (FP)

	FP setting		Total (*N* = 51)	*p*-Value
	Preoperative FP (*N* = 32, 62.7%)	Postoperative FP (*N* = 19, 37.3%)		
Age at diagnosis, mean (SD)*	33.2 (4.38)	35.4 (5.24)	34.0 (4.75)	0.1338^1^
BMI, median (IQR)*	24.5 (22.5, 29.7)	25.2 (22.9, 29.0)	24.9 (22.8, 29.5)	0.5995^2^
*Race and ethnicity*				0.8745^3^
Non-Hispanic white	17 (53.13%)	12 (63.16%)	29 (56.86%)	
Non-Hispanic Black	4 (12.50%)	2 (10.53%)	6 (11.76%)	
Asian	3 (9.38%)	2 (10.53%)	5 (9.80%)	
Hispanic	7 (21.88%)	2 (10.53%)	9 (17.65%)	
Other	1 (3.13%)	1 (5.26%)	2 (3.92%)	
*Histology*				0.0293^3^
DCIS	0 (0.00%)	2 (10.53%)	2 (3.92%)	
Ductal	31 (96.88%)^a^	14 (73.68%)^b^	45 (88.24%)	
Lobular	1 (3.13%)	2 (10.53%)	3 (5.88%)	
Other	0 (0.00%)	1 (5.26%)	1 (1.96%)	
*Clinical stage, AJCC 8th Ed.*				0.0735^3^
I	9 (28.13%)	5 (29.41%)	14 (28.57%)	
II	17 (53.13%)	10 (58.82%)	27 (55.10%)	
III	6 (18.75%)	0 (0.00%)	6 (12.24%)	
IV	0 (0.00%)	2 (11.76%)	2 (4.08%)	
*Missing*	*0*	*2*	*2*	
*Clinical N category*				0.0231^3^
N0	15 (46.88%)^a^	15 (78.95%)^b^	30 (58.82%)	
N1	16 (50.00%)^a^	3 (15.79%)^b^	19 (37.25%)	
N3	1 (3.13%)	1 (5.26%)	2 (3.92%)	
*Grade*				0.0060^3^
1	0 (0.00%)	2 (11.76%)	2 (4.35%)	
2	7 (24.14%)^a^	9 (52.94%)^b^	16 (34.78%)	
3	22 (75.86%)^a^	6 (35.29%)^b^	28 (60.87%)	
*Missing*	*3*	*2*	*5*	
*PR status*				0.2313^3^
Negative	7 (21.88%)	1 (5.26%)	8 (15.69%)	
Positive	25 (78.13%)	18 (94.74%)	43 (84.31%)	
*HER2 status*				0.0147^3^
Negative	14 (43.75%)^a^	14 (82.35%) ^b^	28 (57.14%)	
Positive	18 (56.25%)^a^	3 (17.65%)^b^	21 (42.86%)	
*Missing*	*0*	*2*	*2*	

*n = 2 missing

^1^Equal variance two sample* t*-test; ^2^Kruskal–Wallis *p*-value; ^3^Fisher exact *p*-value

^a,b^Post hoc standardized Pearson residual analysis indicated a statistically significant difference between variable levels.

### Treatment Characteristics and Response

All patients who received NST underwent preoperative FP (*N* = 30). All patients who received NST experienced treatment response with a mean tumor volume reduction of 79.2% (SD 22.6) from pre-NST to post-NST imaging (28 ultrasound, 3 magnetic resonance imaging, 1 computed tomography). pCR rate was 25.8%. Median time from diagnosis to NST initiation was 46.0 days (IQR 37.3–79.5 days) for patients in the preoperative FP cohort. Median time from diagnosis to surgery was 32.0 days (IQR 21.5–66.0 days) for the postoperative FP cohort. There were no significant differences between preoperative FP and postoperative FP cohorts with respect to the type of breast or axillary surgery (Table 2).

**Table 2:** Treatment characteristics and response, by timing of fertility preservation (FP)

	FP setting		Total (*N* = 51)	*p*-Value^ψ^
	Preoperative FP (*N* = 32, 62.7%)	Postoperative FP (*N* = 19, 37.3%)		
*Neoadjuvant systemic therapy, n (%)*				< 0.0001
No	2 (6.25%)^a^	19 (100.00%)^b^	21 (41.18%)	
Yes	30 (93.75%)^a^	0 (0.00%)^b^	30 (58.82%)	
*Pathological complete response, n (%)*				0.0799
No	25 (78.13%)	15 (100.00%)	40 (85.11%)	
Yes	7 (21.88%)	0 (0.00%)	7 (14.89%)	
*Missing*	*0*	*4*	*4*	
*Breast surgery type, n (%)*				0.0847
Segmental mastectomy	12 (37.50%)	13 (72.22%)	25 (50.00%)	
Skin-sparing mastectomy	15 (46.88%)	3 (16.67%)	18 (36.00%)	
Total mastectomy	3 (9.38%)	1 (5.56%)	4 (8.00%)	
Nipple-sparing mastectomy	1 (3.13%)	0 (0.00%)	1 (2.00%)	
Modified radical mastectomy	1 (3.13%)	1 (5.56%)	2 (4.00%)	
*Missing*	*0*	*1*	*1*	
*Axillary surgery type, n (%)*				0.2796
None	0 (0.00%)	1 (6.25%)	1 (2.08%)	
SLNB	15 (46.88%)	10 (62.50%)	25 (52.08%)	
ALND	15 (46.88%)	5 (31.25%)	20 (41.67%)	
TAD	2 (6.25%)	0 (0.00%)	2 (4.17%)	
*Missing*	*0*	*3*	*3*	

^ψ^Fisher exact *p*-value

^a,b^Post hoc standardized Pearson residual analysis indicated a statistically significant difference between variable levels.

### Fertility Preservation Outcomes

Overall, 51 patients underwent 58 COS cycles (Table 3). A majority of patients underwent standard start stimulation during the early follicular phase of the menstrual cycle (44, 86.3%). Patients undergoing preoperative FP were more likely to utilize a random start protocol (14.8% versus 0%, *p* = 0.04) and to receive concurrent letrozole to lower estradiol levels during stimulation (82.1% versus 52.4%, *p* = 0.04) compared with those undergoing postoperative FP. Peak estradiol levels did not differ significantly by timing of COS (*p* = 0.28). Median number of mature oocytes retrieved, oocytes cryopreserved, and embryos cryopreserved were comparable between the preoperative versus postoperative FP cohorts (Supplementary Table 1).

**Table 3:** Neoadjuvant systemic therapy subgroup: baseline characteristics, by receipt of fertility preservation (study cohort versus propensity-score matches)

	Study cohort		Total (*N* = 60)
	Fertility preservation (*N* = 30)	Matched control (*N* = 30)	
*Age at diagnosis*			
*N*	30	30	60
Mean (SD)	33.4 (4.33)	33.7 (4.66)	33.5 (4.46)
Minimum, maximum	26.4, 44.2	24.8, 45.0	24.8, 45.0
*Race and ethnicity, n (%)*			
Non-Hispanic white	15 (50.00%)	18 (60.00%)	33 (55.00%)
Non-Hispanic Black	4 (13.33%)	4 (13.33%)	8 (13.33%)
Asian	3 (10.00%)	5 (16.67%)	8 (13.33%)
Hispanic	7 (23.33%)	3 (10.00%)	10 (16.67%)
Other	1 (3.33%)	0 (0.00%)	1 (1.67%)
*BMI*			
*N*	30	30	60
Median	23.9	25.0	24.5
IQR	7.0	8.0	7.5
Q1, Q3	22.5, 29.5	22.0, 30.0	22.2, 29.7
Minimum, maximum	20.3, 36.8	18.0, 43.0	18.0, 43.0
*Clinical T category, n (%)*			
T1	8 (26.67%)	4 (13.33%)	12 (20.00%)
T2	13 (43.33%)	17 (56.67%)	30 (50.00%)
T3	9 (30.00%)	9 (30.00%)	18 (30.00%)
*Clinical N category, n (%)*			
N0	13 (43.33%)	17 (56.67%)	30 (50.00%)
N1	16 (53.33%)	9 (30.00%)	25 (41.67%)
N2	0 (0.00%)	2 (6.67%)	2 (3.33%)
N3	1 (3.33%)	2 (6.67%)	3 (5.00%)
*Clinical stage, AJCC 8th, n (%)*			
I	7 (23.33%)	3 (10.00%)	10 (16.67%)
II	17 (56.67%)	23 (76.67%)	40 (66.67%)
III	6 (20.00%)	4 (13.33%)	10 (16.67%)
*PR status, n (%)*			
Negative	7 (23.33%)	6 (20.00%)	13 (21.67%)
Positive	23 (76.67%)	24 (80.00%)	47 (78.33%)
*HER2 status, n (%)*			
Negative	13 (43.33%)	12 (40.00%)	25 (41.67%)
Positive	17 (56.67%)	18 (60.00%)	35 (58.33%)
*Receptor subtype, n (%)*			
HR+/HER2+	17 (56.67%)	18 (60.00%)	35 (58.33%)
HR+/HER2−	13 (43.33%)	12 (40.00%)	25 (41.67%)
*Percent tumor volume decrease pre- to post-NAC*			
*N*	27	27	54
Median	85.1	90.3	88.9
IQR	18.0	21.2	17.7
Q1, Q3	74.8, 92.8	78.8, 100.0	78.8, 96.4
Minimum, maximum	4.4, 100.0	62.4, 100.0	4.4, 100.0
*Missing	*3*	*3*	*3*
*Days from diagnosis to neoadjuvant chemotherapy start*			
*N*	30	30	60
Median	48.0	29.5	40.5
IQR	33.0	17.0	27.5
Q1, Q3	39.0, 72.0	24.0, 41.0	28.0, 55.5
Minimum, maximum	13.0, 142.0	11.0, 66.0	11.0, 142.0

*Control values were missing for three cases; therefore, all three pairs were excluded

### Survival Outcomes by Timing of Ovarian Stimulation

**Fig. 1:**
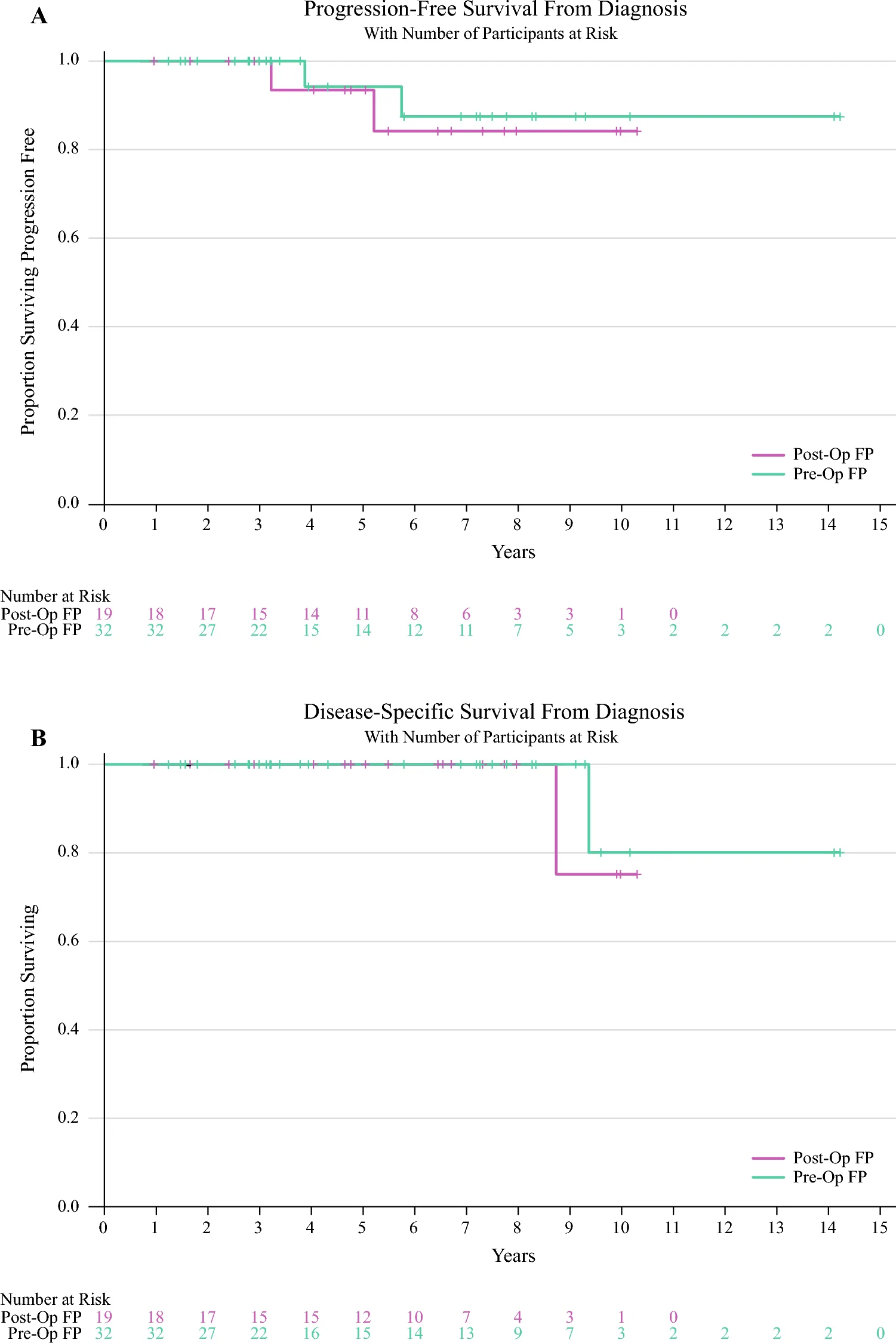
Progression-free survival (**A**) and disease-specific survival (**B**) by timing of fertility preservation (FP)

There were no significant differences in PFS or DSS by timing of FP (Fig. 1). Median follow-up for censored observations was 5.5 years (95% CI 3.4–7.2) for PFS and 5.5 years (95% CI 3.4–7.3) for DSS. The 5-year estimated PFS was 94.1% (95% CI 65.0–99.2%) for the preoperative FP cohort compared with 93.3% (95% CI 61.3–99.0%) for the postoperative FP cohort (log-rank test *χ*^2^(1) = 0.12, *p* = 0.73). The 10-year estimated DSS was 80% (95% CI 20.4–96.9%) for the preoperative FP cohort compared with 75.0% (95% CI 12.8–96.1%) for the postoperative FP cohort (log rank test *χ*^2^(1) = 0.15, *p* = 0.70). Overall, there were two breast-cancer-specific deaths, four recurrences (one local regional, three distant), and two disease progression events among three preoperative and three postoperative FP patients at last follow-up. Importantly, there were no events of disease progression during the neoadjuvant period among patients who underwent preoperative FP.

### NST Subcohort Analysis with Propensity Score Matching

A total of 30 patients who received NST and underwent preoperative FP were matched 1:1 on the basis of age, HER2 status, and clinical stage from an institutional cohort of patients with ER+ breast cancer who underwent NST but did not undergo FP (*N* = 308 candidate matches, Supplementary Fig. 1). Baseline demographics, clinicopathologic, and treatment characteristics were comparable between the preoperative FP cohort versus the matched cohort (Table 3). The preoperative FP cohort experienced a significantly longer time from diagnosis to NST initiation compared with the matched cohort (median 48 versus 29.5 days, *p* < 0.0001). All patients experienced treatment response with similar reductions in tumor volume from pre-NST to post-NST imaging: median 85.1% (IQR 74.8–92.8) versus 90.3% (78.8–100.0),* p *= 0.14.

**Fig. 2:**
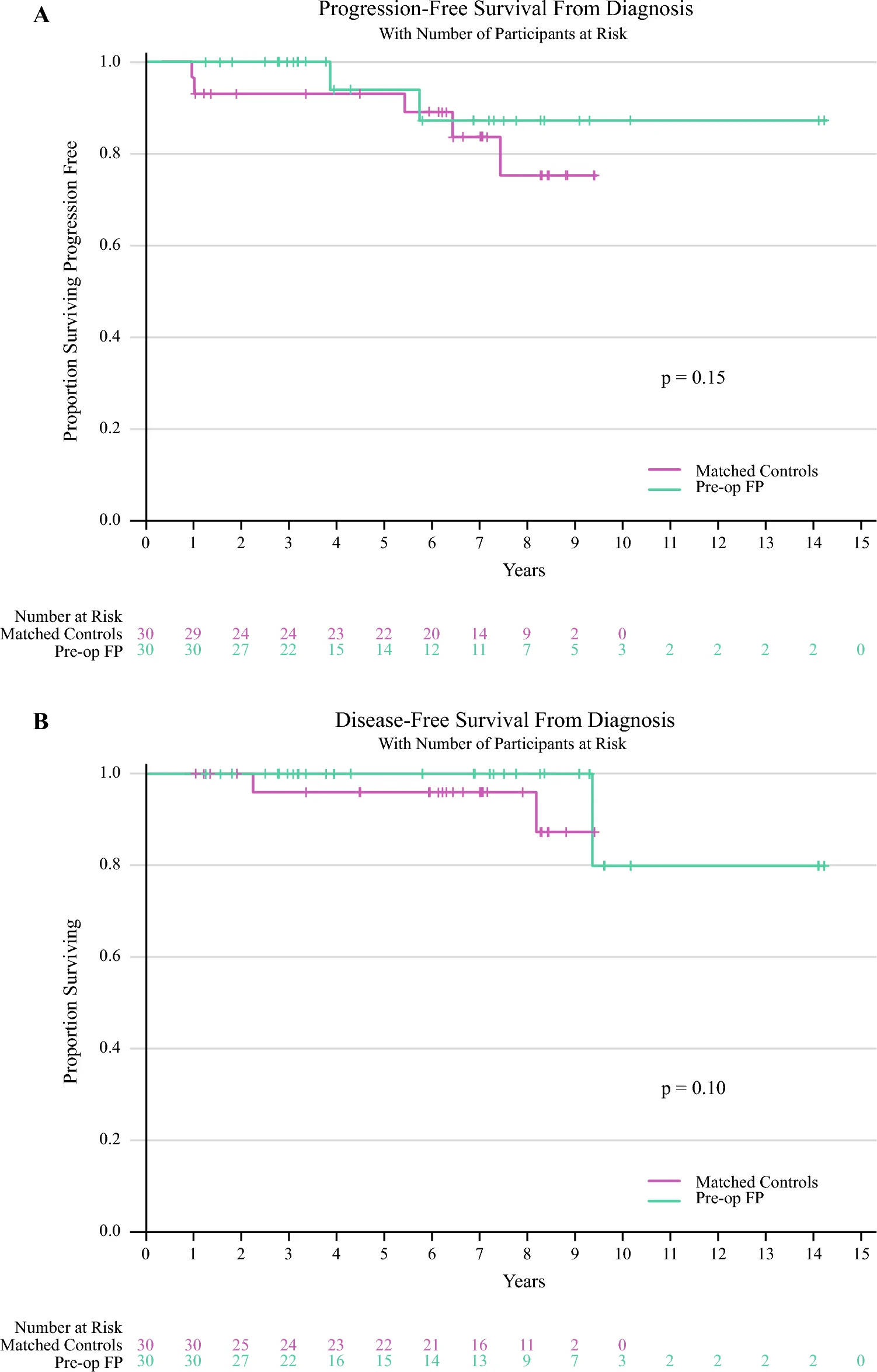
Neoadjuvant systemic therapy subgroup: progression-free survival (**A**) and disease-specific survival (**B**) by receipt of fertility preservation (study cohort versus propensity-score matches)

At an overall median follow-up time of 6.9 years (95% CI 4.5–7.3), there were no significant differences in PFS or DSS between the preoperative FP and matched cohort (Fig. 2). The 5-year estimated PFS was 94.1% (95% CI 65.0–99.2) for the preoperative FP cohort versus 93.3% (95% CI 75.9–98.3) for the matched cohort (stratified log-rank test *χ*^2^(29) = 36.94, *p* = 0.15). The 5-year estimated DSS was 100% (95% CI not applicable) for the preoperative FP cohort compared with 96.0% (95% CI 74.8–99.4%) for the matched cohort (stratified log-rank test *χ*^2^(25) = 34.35, *p* = 0.10). In total, there were seven events of progression (*N* = 2 preoperative FP cohort versus *N* = 5 matched cohort) and three deaths on last follow-up (*N* = 1 preoperative FP cohort versus *N* = 2 matched cohort).

## Discussion

In this cohort of patients who underwent preoperative FP with an in situ ER+ breast cancer, there were no events of disease progression in the neoadjuvant period. Importantly, 5-year survival outcomes were similar to patients with ER+ breast cancer undergoing COS after surgical resection, as well as to propensity score matched patients receiving NST who did not undergo COS. Taken together, our data support the oncologic safety of preoperative COS among patients with ER+ breast cancer who desire FP prior to receiving gonadotoxic NST. While multiple prior studies support the safety of FP after surgical resection and prior to adjuvant systemic therapy,^7–15^ ours is the first to specifically evaluate its safety in the neoadjuvant setting with an in situ ER+ breast tumor.

These results provide much-needed evidence for young women with breast cancer who desire future childbearing during survivorship. Up to 77% of patients with breast cancer report significant levels of psychological distress over the potential loss of fertility as a result of oncologic therapies.^6,26,27^ In a survey of 620 patients less than 40 years of age with early stage breast cancer, 50% expressed concerns over infertility after cancer treatment.^27^ However, only 10% utilized FP strategies prior to treatment initiation.^27^ Fear of disease progression and future risk of recurrence served as major barriers to patient pursuit of FP.^6,27^ Hesitancy over the safety of FP is likewise equally present among physicians.^5,28^ Our results provide reassuring evidence to patients and physicians on the safety of preoperative FP for ER+ breast cancer and may inform future efforts to study this patient population in a larger, prospective fashion.

Although national and international guidelines support FP in patients of reproductive age with breast cancer who will receive gonadotoxic treatments,^3,4^ data supporting the safety of FP in the neoadjuvant setting are limited. Prior studies of FP in patients with breast cancer utilized heterogeneous cohorts with low numbers of patients receiving NST.^8,10,11,13,18,20^ For instance, Kim and colleagues examined 120 patients who underwent oocyte cryopreservation prior to chemotherapy for breast cancer, of whom only 14 underwent FP in the neoadjuvant setting.^13^ There were no differences in recurrence rates and relapse-free survival between FP versus control groups in the overall study cohort and between the pre- versus postoperative FP groups. Similarly, Greer and colleagues reported similar survival outcomes among 123 young women with early stage breast cancer undergoing FP compared with 149 who did not, but only one-third received NST.^19^ A major strength of our study is the use of propensity score matching to carefully evaluate the impact of FP prior to NST. We observed no significant differences in tumor volume reduction, PFS, or DFS among women who did versus did not undergo FP prior to NST after controlling for age, HER2 receptor status, and clinical stage. Furthermore, patients with higher tumor burden, such as those who had clinically node-positive disease, high-grade disease, and HER2-positive disease, were more likely to undergo preoperative FP compared with postoperative FP. Given that patients with increased tumor burden may receive cumulatively greater levels of gonadotoxic therapies, early initiation of FP in the neoadjuvant setting may allow for the most optimal fertility preservation. As the indications for NST continue to expand, particularly among patients with high-risk disease, nodal metastases, and those who desire breast conservation therapy, the need for preoperative FP in the neoadjuvant setting will likely increase, underscoring the impact of our findings.

Another major hesitation with undergoing FP prior to NST is the potential delay in treatment initiation. In our study, median time from diagnosis to NST initiation was 48 days in patients undergoing preoperative FP versus 29.5 days in the propensity score matched cohort not undergoing COS. Among the three other studies in literature that have examined time to treatment after FP, two demonstrated similar time to NST initiation while one demonstrated a 10-day delay prior to NST initiation.^11,18,19^ Nonetheless, our overall range of 29.5–48 days is consistent with the range of 26–41.5 days reported in these studies. The modest delay in treatment initiation observed in our preoperative FP cohort may be attributable to the fact that many patients travel to seek evaluation at our tertiary cancer center and subsequently elect to receive neoadjuvant treatment locally with community medical oncologists. However, this delay in treatment initiation did not translate to a clinically meaningful difference as 5-year survival outcomes were comparable between the preoperative FP cohort and the propensity score matched cohort. Furthermore, prior work evaluating time to NST initiation among patients with breast cancer demonstrates that while delays in treatment > 60 days from diagnosis translated to an increased risk of death, those within 31–60 days demonstrated similar outcomes.^29^ As such, our findings suggest that any modest delays in cancer care to accommodate FP procedures are unlikely to impact oncologic outcomes and therefore should not serve as a barrier to pursuing FP in the neoadjuvant setting.

Fear of disease progression is another major barrier to FP utilization. This is of particular relevance for patients with ER+ breast cancer planned for NST as COS will expose their in situ, hormone-sensitive tumor to supraphysiologic estrogen levels that may promote tumor growth and metastasis. Only one prior study has specifically examined FP among patients with breast cancer planned for NST, with few having ER+ disease. In a cohort of patients undergoing FP before NST in the I-SPY2 trial, Chien and colleagues reported similar 5-year PFS among 34 patients—including 16 with hormone receptor-positive disease—who underwent FP versus 48 patients who did not.^11^ Our results confirm these findings in a higher-risk cohort restricted to patients with ER+ disease, those most susceptible to estrogen-induced tumor growth and dissemination in the preoperative setting. Concomitant use of aromatase inhibitors such as letrozole and anastrozole or selective estrogen receptor modulators such as tamoxifen serves as one method to counteract the supraphysiologic levels of estrogen during ovarian stimulation.^30^ In our study cohort, more than 80% of patients received concomitant letrozole suppression. However, even among the five remaining patients who did not receive letrozole, there were no events of disease progression with the primary tumor in situ.

Beyond survival outcomes, timing of FP did not appear to impact success of ovarian stimulation. Specifically, median mature oocytes retrieved and cryopreserved were similar between the preoperative versus postoperative FP cohorts. Additionally, the range of 10–12 mature oocytes retrieved and oocytes cryopreserved are in line with ranges reported in previous studies.^10,14,31^ While FP success may differ by fertility center practices and patient characteristics, these results should provide reassurance to patients that FP can be safely pursued in the neoadjuvant setting without compromising oncologic or fertility outcomes.

As with any study, our data should be interpreted in the setting of its limitations. First, this was a small retrospective cohort study at a tertiary cancer center and as such, may have limited generalizability. Second, given that only seven patients experienced progression in the propensity score matching analysis, our ability to detect a statistically significant association may have been limited by low power. Furthermore, the small number of events restricted our capacity to control for potential confounders and other covariates in multivariable analysis. For instance, we were unable to examine impact of age, body mass index (BMI), and HER2 receptor status on recurrence rates and survival outcomes given small sample size. However, propensity score analysis allowed for matching by age, HER2 status, and clinical stage among those undergoing NST in the presence or absence of FP. Third, given the retrospective design of our study, we were unable to evaluate whether decision-making about FP was influenced by concerns about potential delay in NST receipt. Fourth, while we were able to compare pre- and post-NST tumor volumes and report pCR rates, we were unable to report residual cancer burden (RCB) as pathology reports from earlier in the study period were not standardized to report all RCB elements. Lastly, given the propensity of ER+ breast cancer to exhibit late recurrences, longer-term follow-up data beyond 5 years will provide more definitive evidence on the impact of FP timing.

## Conclusions

In this cohort of women with breast cancer, FP with COS in the setting of an in situ ER+ tumor was not significantly associated with increased risks of disease progression or mortality. These findings support the oncologic safety of preoperative COS in this population. As many of these patients require gonadotoxic NST and desire future childbearing, these results hold important implications for oncofertility counseling and survivorship-focused treatment decisions.

## Supplementary Information

Below is the link to the electronic supplementary material.

Supplementary file1 (DOCX 212 KB)

## Abbreviations

COS: Controlled ovarian stimulation
FP: Fertility preservation
ER: Estrogen receptor
PR: Progesterone receptor
HER2: Human epidermal growth factor receptor 2
pCR: Pathologic complete response
NST: Neoadjuvant systemic therapy
PFS: Progression-free survival
DSS: Disease-specific survival
AJCC: American joint committee on cancer

## References

[1] Miller KD, Fidler-Benaoudia M, Keegan TH, Hipp HS, Jemal A, Siegel RL. Cancer statistics for adolescents and young adults, 2020. *CA Cancer J Clin*. 2020;70(6):443–59. 10.3322/caac.21637. 32940362

[2] American Cancer Society. Key Statistics for Breast Cancer. January 17, 2024. Accessed April 28, 2024. https://www.cancer.org/cancer/types/breast-cancer/about/how-common-is-breast-cancer.html#:~:text=About%20310%2C720%20new%20cases%20of,will%20die%20from%20breast%20cancer.

[3] Oktay K, Harvey BE, Partridge AH, et al. Fertility preservation in patients with cancer: ASCO clinical practice guideline update. *J Clin Oncol*. 2018;36(19):1994–2001. 10.1200/JCO.2018.78.1914. 29620997

[4] Lambertini M, Peccatori FA, Demeestere I, et al. Fertility preservation and post-treatment pregnancies in post-pubertal cancer patients: ESMO clinical practice guidelines. *Ann Oncol*. 2020;31(12):1664–78. 10.1016/j.annonc.2020.09.006. 32976936

[5] Lambertini M, Di Maio M, Pagani O, et al. The BCY3/BCC 2017 survey on physicians’ knowledge, attitudes and practice towards fertility and pregnancy-related issues in young breast cancer patients. *Breast*. 2018;42:41–9. 10.1016/j.breast.2018.08.099. 30170202

[6] Partridge AH, Gelber S, Peppercorn J, et al. Web-based survey of fertility issues in young women with breast cancer. *J Clin Oncol*. 2004;22(20):4174–83. 10.1200/JCO.2004.01.159. 15483028

[7] Arecco L, Perachino M, Damassi A, et al. Burning questions in the oncofertility counseling of young breast cancer patients. *Breast Cancer* (*Auckl*). 2020;14. https://api.semanticscholar.org/CorpusID:221763048 10.1177/1178223420954179 PMC7476336 32952399

[8] Rodriguez-Wallberg KA, Eloranta S, Krawiec K, Lissmats A, Bergh J, Liljegren A. Safety of fertility preservation in breast cancer patients in a register-based matched cohort study. *Breast Cancer Res Treat*. 2018;167(3):761–9. 10.1007/s10549-017-4555-3. PMC5807458 29098528

[9] Arecco L, Blondeaux E, Bruzzone M, et al. Safety of fertility preservation techniques before and after anticancer treatments in young women with breast cancer: a systematic review and meta-analysis. *Human Reprod*. 2022;37(5):954–68. 10.1093/humrep/deac035. PMC9071231 35220429

[10] Moravek MB, Confino R, Lawson AK, et al. Predictors and outcomes in breast cancer patients who did or did not pursue fertility preservation. *Breast Cancer Res Treat*. 2021;186(2):429–37. 10.1007/s10549-020-06031-4. 33392838

[11] Chien AJ, Chambers J, Mcauley F, et al. Fertility preservation with ovarian stimulation and time to treatment in women with stage II–III breast cancer receiving neoadjuvant therapy. *Breast Cancer Res Treat*. 2017;165(1):151–9. 10.1007/s10549-017-4288-3. 28503722

[12] Azim AA, Costantini-Ferrando M, Oktay K. Safety of fertility preservation by ovarian stimulation with letrozole and gonadotropins in patients with breast cancer: a prospective controlled study. *J Clin Oncol*. 2008;26(16):2630–5. 10.1200/JCO.2007.14.8700. 18509175

[13] Kim J, Turan V, Oktay K. Long-term safety of letrozole and gonadotropin stimulation for fertility preservation in women with breast cancer. *J Clin Endocrinol Metab*. 2016;101(4):1364–71. 10.1210/jc.2015-3878. PMC4880171 26751194

[14] Oktay K. Further evidence on the safety and success of ovarian stimulation with letrozole and tamoxifen in breast cancer patients undergoing in vitro fertilization to cryopreserve their embryos for fertility preservation. *J Clin Oncol*. 2005;23(16):3858–9. 10.1200/JCO.2005.04.011. 15911867

[15] Marklund A, Lekberg T, Hedayati E, et al. Relapse rates and disease-specific mortality following procedures for fertility preservation at time of breast cancer diagnosis. *JAMA Oncol*. 2022;8(10):1438–46. 10.1001/jamaoncol.2022.3677. PMC9412846 36006625

[16] Joo B, Park S, An B, Kim K, Moon S, Moon H. Serum estradiol levels during controlled ovarian hyperstimulation influence the pregnancy outcome of in vitro fertilization in a concentration-dependent manner. *Fertil Steril*. 2009;93:442–6. 10.1016/j.fertnstert.2009.02.066. 19394001

[17] Platet N, Cathiard AM, Gleizes M, Garcia M. Estrogens and their receptors in breast cancer progression: a dual role in cancer proliferation and invasion. *Crit Rev Oncol Hematol*. 2004;51(1):55–67. 10.1016/j.critrevonc.2004.02.001. 15207254

[18] Letourneau JM, Sinha N, Wald K, et al. Random start ovarian stimulation for fertility preservation appears unlikely to delay initiation of neoadjuvant chemotherapy for breast cancer. *Human Reprod*. 2017;32(10):2123–9. 10.1093/humrep/dex276. 28938748

[19] Greer AC, Lanes A, Poorvu PD, et al. The impact of fertility preservation on the timing of breast cancer treatment, recurrence, and survival. *Cancer*. 2021;127(20):3872–80. 10.1002/cncr.33601. 34161610

[20] Muñoz E, Domingo J, De Castro G, et al. Ovarian stimulation for oocyte vitrification does not modify disease-free survival and overall survival rates in patients with early breast cancer. *Reprod Biomed Online*. 2019;39(5):860–7. 10.1016/j.rbmo.2019.07.003. 31564650

[21] Rosenbaum PR, Rubin DB. The central role of the propensity score in observational studies for causal effects. *Biometrika*. 1983;70(1):41–55. 10.2307/2335942.

[22] McDonald RJ, McDonald JS, Kallmes DF, Carter RE. Behind the numbers: propensity score analysis—a primer for the diagnostic radiologist. *Radiology*. 2013;269(3):640–5. 10.1148/radiol.13131465. 24261493

[23] Austin PC. A critical appraisal of propensity-score matching in the medical literature between 1996 and 2003. *Stat Med*. 2008;27(12):2037–49. 10.1002/sim.3150. 18038446

[24] Stuart EA, Cole SR, Bradshaw CP, Leaf PJ. The use of propensity scores to assess the generalizability of results from randomized trials. *J R Stat Soc Ser A Stat Soc*. 2011;174(2):369–86. 10.1111/j.1467-985X.2010.00673.x. PMC4051511 24926156

[25] Rubin DB. Using propensity scores to help design observational studies: application to the tobacco litigation. *Health Serv Outcomes Res Methodol*. 2001;2(3):169–88. 10.1023/A:1020363010465.

[26] Lambertini M, Anserini P, Fontana V, et al. The PREgnancy and FERtility (PREFER) study: an Italian multicenter prospective cohort study on fertility preservation and pregnancy issues in young breast cancer patients. *BMC Cancer*. 2017;17(1):346. 10.1186/s12885-017-3348-8. PMC5437418 28526012

[27] Ruddy KJ, Gelber SI, Tamimi RM, et al. Prospective study of fertility concerns and preservation strategies in young women with breast cancer. *J Clin Oncol*. 2014;32(11):1151–6. 10.1200/JCO.2013.52.8877. PMC4164759 24567428

[28] Biglia N, Torrisi R, D’Alonzo M, Codacci Pisanelli G, Rota S, Peccatori FA. Attitudes on fertility issues in breast cancer patients: an Italian survey. *Gynecol Endocrinol*. 2015;31(6):458–64. 10.3109/09513590.2014.1003293. 25982361

[29] de Melo Gagliato D, Lei X, Giordano SH, et al. Impact of delayed neoadjuvant systemic chemotherapy on overall survival among patients with breast cancer. *Oncologist*. 2020;25(9):749–57. 10.1634/theoncologist.2019-0744. PMC7485349 32431013

[30] Fatum M, McVeigh E, Child T. The case for aromatase inhibitors use in Oncofertility patients. Should aromatase inhibitors be combined with gonadotropin treatment in Breast Cancer patients undergoing ovarian stimulation for fertility preservation prior to chemotherapy? *A debate Hum Fertil*. 2013;16(4):235–40. 10.3109/14647273.2013.800650. 23863119

[31] Letourneau JM, Wald K, Sinha N, et al. Fertility preservation before breast cancer treatment appears unlikely to affect disease-free survival at a median follow-up of 43 months after fertility-preservation consultation. *Cancer*. 2020;126(3):487–95. 10.1002/cncr.32546. 31639215

