# Supplementary material for: Oncologic Safety of Preoperative Controlled Ovarian Stimulation for Fertility Preservation Among Women with Estrogen Receptor Positive Breast Cancer
Source: Ann Surg Oncol. 2026 Jul 5;33(11):9899–909. doi: 10.1245/s10434-026-20156-8 (PMC13630988; doi:10.1245/s10434-026-20156-8)
Supplement: Supplementary file 1 [file 10434_2026_20156_MOESM1_ESM.docx]

**Online-Only Supplement**

Table S1. Controlled Ovarian Stimulation (COS) Outcomes

Figure S1. Assessment of Propensity Score Matching Balance

**Table S1. Controlled Ovarian Stimulation (COS) Outcomes**

| **Variable** | **Pre-operative COS (N=32, 61.5%)**  **35 Cycles** | **Post-operative COS (N=19, 37.3%)**  **23 Cycles** | **p-value** | |
| --- | --- | --- | --- | --- |
| Protocol type |  |  | **0.038** | |
| Standard start | 23 (85.2%) | 21 (95.5%) |  | |
| Luteal start | 0 (0.0%) | 1 (4.5%) |  | |
| Random start | 4 (14.8%) | 0 (0.0%) |  | |
| Missing | 8 | 1 |  |  |
| Peak E2 level (pg/ml), median (IQR) | 560.0 (334.4-1299.0) | 687.5 (442.5-1837.5) | 0.284 | |
| Letrozole |  |  |  | |
| Yes | 23 (82.1%) | 11 (52.4%) | **0.039** | |
| No | 5 (17.9%) | 10 (47.6%) |  | |
| Missing | 7 | 2 |  |  |
| Median mature oocytes retrieved (IQR) | 10.0  (5.0-16.0) | 12.0  (5.0-14.0) | 0.713 | |
| Median oocytes cryopreserved (IQR) | 10.0 (5.0-14.0) | 12.0 (11.5-15.5) | 0.114 | |
| Median embryos cryopreserved (IQR) | 5.0 (2.0-8.0) | 3.0 (1.0-5.0) | 0.144 | |

*Abbreviations: COS = controlled ovarian stimulation, IQR = interquartile range*

**Figure S1. Assessment of Propensity Score Matching Balance**

**Figure S1.** This figure displays the distribution of the logit of propensity scores for 1) all patients with ER+ breast cancer who underwent neoadjuvant chemotherapy, 2) observations within the support region as defined by patients who underwent fertility preservation before neoadjuvant chemotherapy, and 3) matched controls. Propensity score logits were more similar between patients who received fertility preservation before neoadjuvant chemotherapy (n = 30) and their matched controls (n = 30). Means are shown by open circles or plus signs for controls, with the box indicating the interquartile range and the center line representing the median. Whiskers extend to the minimum and maximum values.
